# Nicaraven Attenuates Radiation-Induced Injury in Hematopoietic Stem/Progenitor Cells in Mice

**DOI:** 10.1371/journal.pone.0060023

**Published:** 2013-03-29

**Authors:** Miho Kawakatsu, Yoshishige Urata, Ryo Imai, Shinji Goto, Yusuke Ono, Noriyuki Nishida, Tao-Sheng Li

**Affiliations:** 1 Department of Stem Cell Biology, Nagasaki University Graduate School of Biomedical Sciences, Nagasaki, Japan; 2 Department of Molecular Microbiology and Immunology, Nagasaki University Graduate School of Biomedical Sciences, Nagasaki, Japan; Indian Institute of Toxicology Reserach, India

## Abstract

Nicaraven, a chemically synthesized hydroxyl radical-specific scavenger, has been demonstrated to protect against ischemia-reperfusion injury in various organs. We investigated whether nicaraven can attenuate radiation-induced injury in hematopoietic stem/progenitor cells, which is the conmen complication of radiotherapy and one of the major causes of death in sub-acute phase after accidental exposure to high dose radiation. C57BL/6 mice were exposed to 1 Gy γ-ray radiation daily for 5 days in succession (a total of 5 Gy), and given nicaraven or a placebo after each exposure. The mice were sacrificed 2 days after the last radiation treatment, and the protective effects and relevant mechanisms of nicaraven in hematopoietic stem/progenitor cells with radiation-induced damage were investigated by *ex vivo* examination. We found that post-radiation administration of nicaraven significantly increased the number, improved the colony-forming capacity, and decreased the DNA damage of hematopoietic stem/progenitor cells. The urinary levels of 8-oxo-2′-deoxyguanosine, a marker of DNA oxidation, were significantly lower in mice that were given nicaraven compared with those that received a placebo treatment, although the levels of intracellular and mitochondrial reactive oxygen species in the bone marrow cells did not differ significantly between the two groups. Interestingly, compared with the placebo treatment, the administration of nicaraven significantly decreased the levels of the inflammatory cytokines IL-6 and TNF-α in the plasma of mice. Our data suggest that nicaraven effectively diminished the effects of radiation-induced injury in hematopoietic stem/progenitor cells, which is likely associated with the anti-oxidative and anti-inflammatory properties of this compound.

## Introduction

Exposure to a high dose of ionizing radiation can directly lead to DNA double-strand breaks that may elicit cell death or stochastic changes [1]. Ionizing radiation is also known to trigger the generation of reactive oxygen species (ROS), which indirectly contribute to radiation-induced damage through the oxidization of biomolecules [2]–[5]. Otherwise, in response to radiation exposure, a robust release of various inflammatory cytokines has also been found to contribute to the subsequent injury of cells or organs [6]–[10]. Therefore, either quickly scavenging the ROS or effectively inhibiting the inflammatory responses is thought to be potential approaches for providing protection against radiation injury. In this regard, previous studies have demonstrated that radiation-induced injury could be attenuated by the administration of antioxidants [11]–[14], and amifostine, a drug with the ability to scavenge ROS, has been used clinically as a cytoprotective adjuvant for patients receiving radiotherapies [15]. However, it is still important to develop new protective and therapeutic drugs that counter the effects of radiation-induced injury due to either therapeutic or accidental exposures.

Nicaraven [N,N¢-(1-methyl-1,2-ethanediyl)bis-3-pyridinecarboxamide], a chemically synthesized hydroxyl radical-specific scavenger [16], has been demonstrated to protect against ischemia-reperfusion injury in various organs, including the brain [17]–[20], liver [21], kidney [22], and heart [23]. Beyond its primary activity as a hydroxyl radical-specific scavenger, nicaraven has also been found to suppress neutrophil infiltration under inflammatory conditions [24], [25]. Based on its well-defined anti-oxidative properties and its likely anti-inflammatory activity, nicaraven may also effectively protect against radiation-induced injury. It has been previously demonstrated that the administration of nicaraven significantly improved the survival of mice that suffered a lethal dose of γ-ray radiation [26]. Nicaraven has also been found to reduce radiation-induced cell death through the inhibition of poly (ADP-ribose) polymerase [27], [28]. However, the protective effect of nicaraven on radiation-induced injury has not yet been well documented, and the relevant mechanism is poorly understood. Using a mouse whole-body γ-ray radiation model, we herein investigated the protective effects and relevant mechanisms of nicaraven on radiation-induced injury in hematopoietic stem/progenitor cells.

## Materials and Methods

### Animals

We used 10- to 12-week-old male C57BL/6 mice (SLC, Japan) for the present study. All experiments were approved by the Institutional Animal Care and Use Committee of Nagasaki University (No. 1108120943), and the animal procedures were performed in accordance with institutional and national guidelines.

### Radiation exposure and nicaraven administration

Whole-body radiation was performed by exposing the mice to γ-rays with a ^137^Cs source at a dose rate of 0.86 Gy/min with a PS-3100SB γ-ray irradiation system (Pony Industry Co., Ltd. Osaka, Japan) [29]. To investigate the protective effect and related mechanisms of nicaraven on radiation-induced injury in hematopoietic stem/progenitor cells, 12 mice were exposed to 1 Gy γ-rays daily for 5 days in succession (a total of 5 Gy) and were then given intraperitoneal injections of nicaraven (100 mg/kg/day, Nicaraven group; n = 6) or saline only (Placebo group; n = 6), respectively, soon after each exposure. The mice were sacrificed 2 days after the last exposure, and samples of urine, blood, and bone marrow cells were collected and used for the following experiments.

### Measurements of nucleated cells and stem cells in the peripheral blood

Samples of heparinized peripheral blood were collected, and the number of nucleated cells in the blood was counted using a Nucleo Counter cell-counting device (Chemotetec A/S, Denmark). To measure the c-kit-positive (c-kit^+^) and CD34-positive (CD34^+^) stem/progenitor cells, we isolated the nucleated cells from the peripheral blood by density gradient centrifugation and then labeled the cells with a PE-conjugated anti-mouse c-kit antibody (eBioscience) or a FITC-conjugated anti-mouse CD34 antibody (BD Bioscience) for 45 min. Respective isotype controls were used as a negative control. After washing, quantitative flow cytometry analysis was performed using a FACSCalibur (Becton Dickinson) (30). We analyzed the acquired data using Cell Quest software (Becton Dickinson).

### Measurement of stem cells in the bone marrow

Bone marrow cells were collected from the femur and tibia, and the mononuclear cells were isolated by density gradient centrifugation [30]. The c-kit^+^ and CD34^+^ cells in the bone marrow mononuclear cells were measured, as described above.

### Colony-forming assay

The colony-forming capacity of the isolated cells was evaluated using mouse methylcellulose complete medium, according to the manufacturer's instructions (R&D System). Briefly, 1×10^5^ peripheral nucleated cells or 3×10^4^ bone marrow mononuclear cells were mixed well with 1 ml of medium, plated in 3-cm culture dishes, and then incubated at 37°C in a 5% CO_2_ incubator. The formation of colonies was observed under a microscope, and the total number of colonies in each dish was counted after 9 days (for bone marrow mononuclear cells) or 12 days (for peripheral blood nucleated cells) of incubation. The mean number of colonies in duplicate assays was used for the statistical analyses.

### Immunocytochemistry

To detect the DNA damage, isolated bone marrow mononuclear cells were seeded on 4-well chamber culture slides (Nalge Nunc International, Roskilde, Denmark) coated with 10 µg/ml fibronectin (Invitrogen) at a density of 3×10^6^ cells/ml in IMDM 1640 medium supplemented with 10% fetal bovine serum (HyClone), 100 units/ml penicillin, and 100 µg/ml streptomycin (Gibco), and incubated at 37°C in 5% CO_2_. The cells were fixed in 1% formaldehyde for 10 min after 7 days of culture. After blocking with 2% bovine serum albumin, the cells were reacted with anti-mouse 53BP1 antibody (Abcam), followed by a FITC-conjugated secondary antibody. The nuclei were stained with Hoechst 33258. The positively stained cells were observed under fluorescence microscopy with 200-fold magnification, and more than 150 cells were counted to calculate the percentage of cells with 53BP1 foci in the nucleus.

### Detection of intracellular and mitochondrial ROS

To elucidate the relevant mechanisms, we measured the intracellular ROS levels based on the oxidation of 5-(and-6)-chloromethyl-2′,7′-dichlorodihydrofluorescein diacetate, acetyl ester (CM-H_2_DCFDA, Molecular Probes Inc.) to form the fluorescent compound 2′,7′-dichlorofluorescein (DCF), as described previously [29], [31]. Briefly, freshly isolated bone marrow mononuclear cells were incubated with 10 µM CM-H_2_DCFDA at 37°C for 30 minutes. After the cells were washed, the fluorescence intensity in the cells was estimated using a FACSCalibur.

The mitochondrial ROS were analyzed with a MitoSOX Red mitochondrial superoxide indicator, as described previously [29]. Briefly, freshly isolated bone marrow stem cells were incubated with 5 µM MitoSOX Red (Molecular Probes Inc.) at 37°C for 30 minutes. After washing, the fluorescence intensity in the cells was estimated using a FACSCalibur.

### Measurements of 8-OHdG, TNF-α, and IL-6 levels in the plasma and urine

We measured the concentrations of 8-oxo-2′-deoxyguanosine (8-OHdG), a marker of DNA oxidation, in the urine and plasma using an ELISA kit (Nikken SEIL Corporation, Shizuoka, Japan), according to the manufacturer's instructions. The concentrations of TNF-α and IL-6 in the plasma were measured with ELISA kits (R&D Systems), as described previously [32]. The mean values of duplicate assays with each sample were used for the statistical analyses.

### Statistical analyses

All results are presented as the means ± SD. Statistical significance between two groups was determined using Mann-Whitney test (Dr. SPSS II, Chicago, IL). Differences were considered significant when p<0.05.

## Results

### Nicaraven attenuated radiation-induced decreases in peripheral blood nucleated cells and stem/progenitor cells

**Figure 1:**
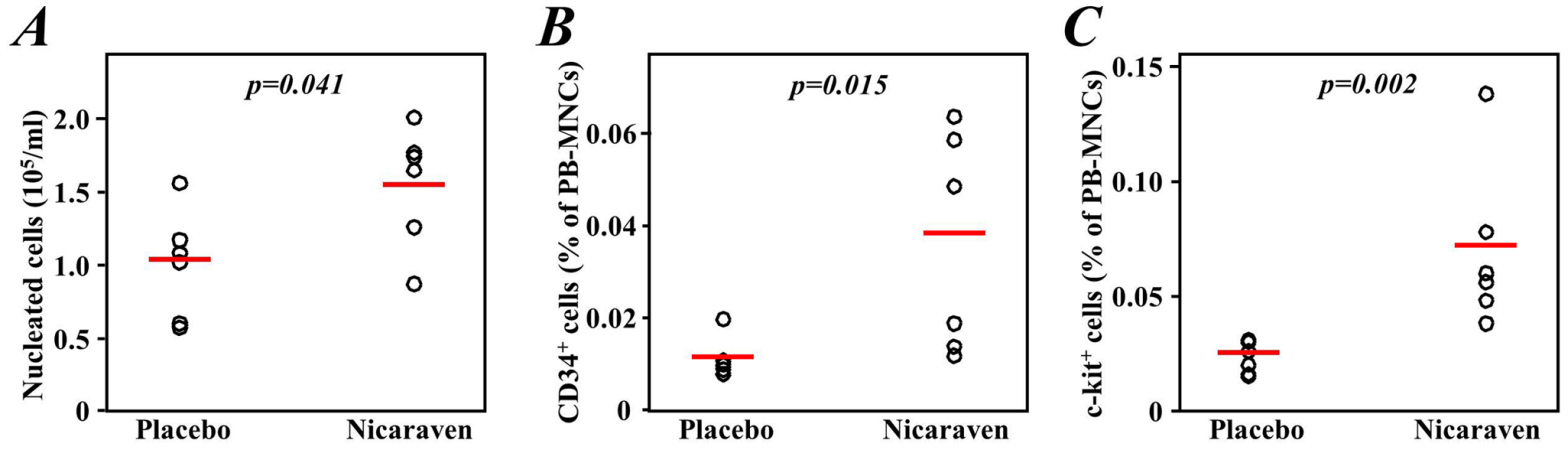
Nucleated cells and stem/progenitor cells in the peripheral blood of mice after treatment. The number of nucleated cells in the peripheral blood (**A**) was directly counted, and c-kit^+^ (**B**) and CD34^+^ stem/progenitor cells (**C**) in the peripheral blood were measured in the fraction of the nucleated cells by flow cytometry. The open circles represent data from each mouse and the red lines indicate median values of each group.

Compared with the placebo treatment, the administration of nicaraven significantly increased the number of nucleated cells in the peripheral blood (p = 0.041; ***Fig. 1A***), although this number was still observed to be as low as approximately 10% of that of the age-matched, non-irradiated healthy mice (16.4±4.2×10^5^/ml, p<0.001 *vs* Placebo and Nicaraven groups). Furthermore, it was shown that the administration of nicaraven also increased the percentages of either c-kit^+^ or CD34^+^ cells in the peripheral blood after radiation exposure (p = 0.015 and p = 0.002, respectively; ***Fig. 1B.C***).

### Nicaraven significantly increased the number and improved the function of hematopoietic stem/progenitor cells in bone marrow

**Figure 2:**
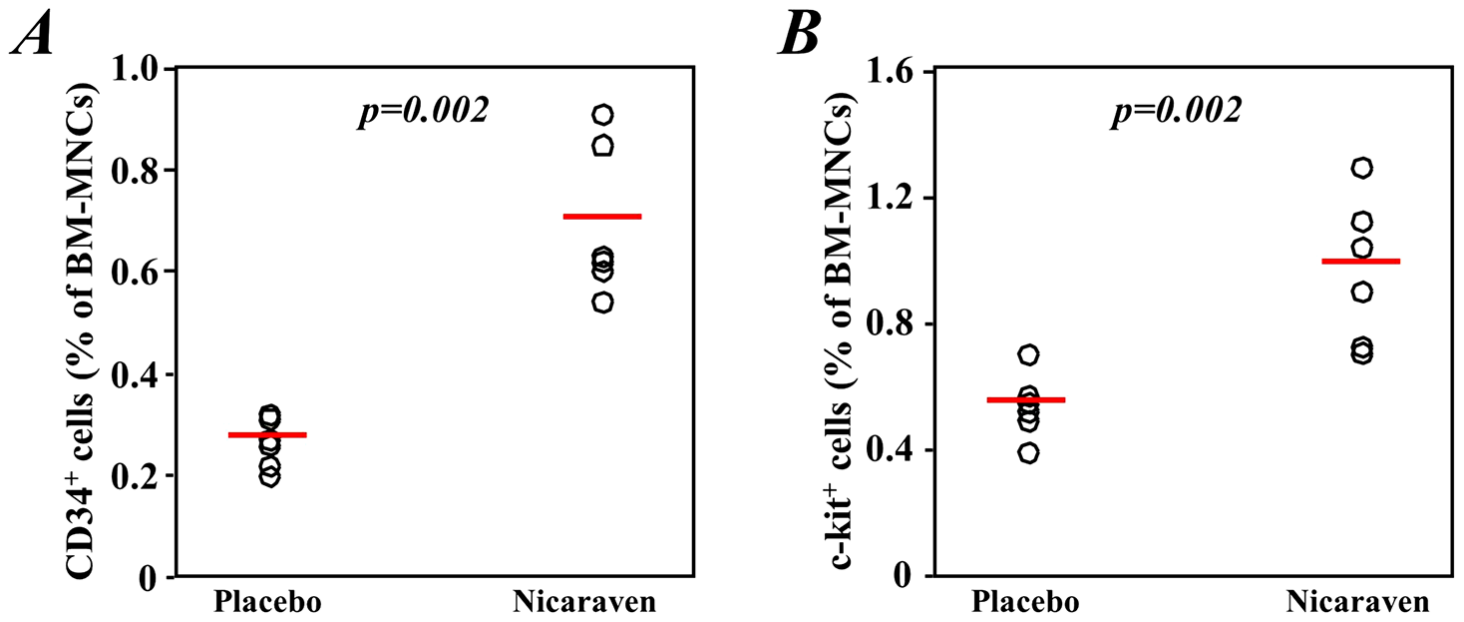
Stem/progenitor cells in the bone marrow of mice after the treatments. Bone marrow mononuclear cells were collected 2 days after the last radiation treatment, and the c-kit^+^ (**A**) and CD34^+^ (**B**) stem/progenitor cells were measured by flow cytometry. The open circles represent data from each mouse and the red lines indicate median values of each group.

The total number of bone marrow mononuclear cells collected each mouse was similar and there was not different between groups. The percentages of c-kit^+^ and CD34^+^ cells were significantly higher in freshly collected bone marrow mononuclear cells from mice that were given nicaraven than those that received a placebo (p = 0.002 and 0.002 *vs.* Placebo group, respectively; ***Fig. 2***). However, the percentage of c-kit^+^ and CD34^+^ stem/progenitor cells in the mice that received nicaraven were still much lower than that of non-irradiated healthy mice (2.72±0.38% and 4.28±0.12%, respectively).

**Figure 3:**
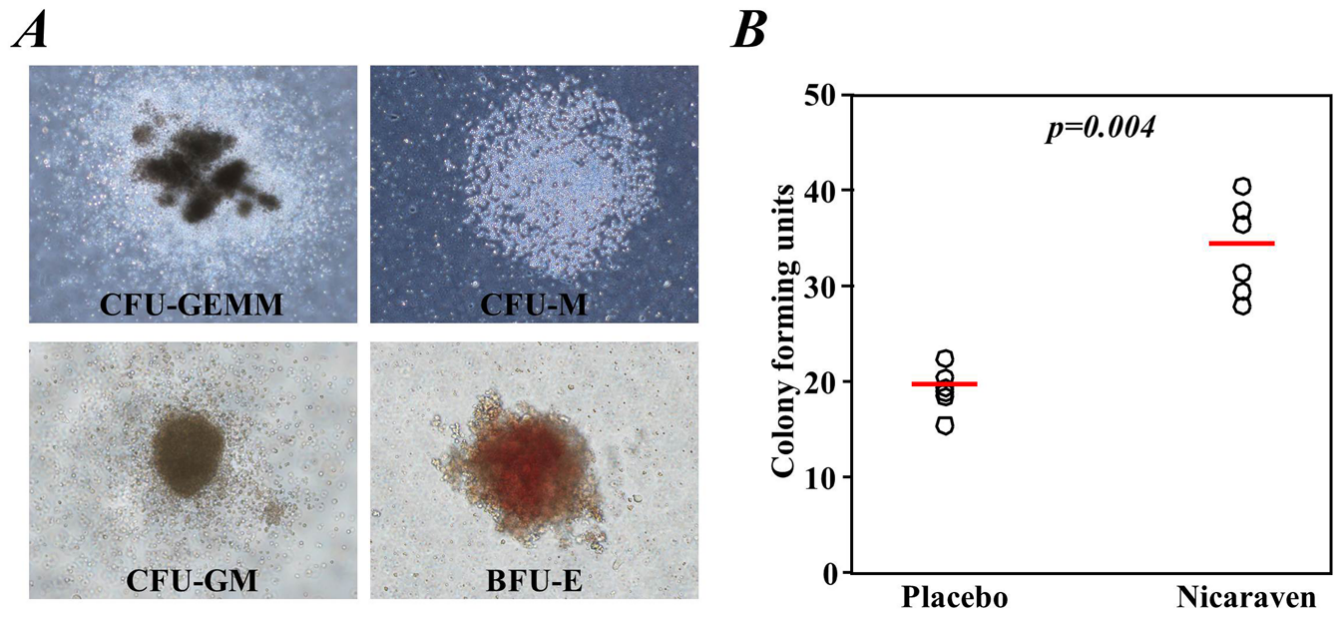
Colony-forming assay. Bone marrow mononuclear cells were collected 2 days after the last radiation treatment. Freshly isolated bone marrow mononuclear cells were mixed in methylcellulose complete medium, and the colony formation was observed under microscopy at 9 days after incubation. **A**) Different types of colonies, including CFU-GEMM, CFU-M, CFU-GM, and BFU-E were clearly formed from the bone marrow cells. **B**) A significantly higher number of total colonies (>50 cells) was formed from the bone marrow cells of the mice that were given nicaraven than those that received placebo. The open circles represent the mean of data from a mouse with duplicate assay. The red lines indicate the median values of each group.

We also performed a colony-forming assay to evaluate the functional impairment of hematopoietic stem/progenitor cells in the bone marrow. The formation of different types of colonies from bone marrow mononuclear cells was clearly observed after 9 days of culture in mouse methylcellulose complete medium (***Fig. 3A***). Although smaller size of colonies was observed in the Placebo group than in the Nicaraven group, there was not obviously difference in the types of colonies (lineage specificity) between groups. By quantitative counting, we found that the total number of colonies formed from bone marrow mononuclear cells was significantly greater in the Nicaraven group than in the Placebo group (p = 0.004, ***Fig. 3B***). However, the numbers of colonies in both the nicaraven- and placebo-treated mice were still less than half of the non-irradiated healthy mice (92.0±8.6, p<0.001). Otherwise, we did not detect any colony formation within 12 days after seeding 1×10^5^ peripheral blood nucleated cells from mice that received either nicaraven or placebo, but approximately 10 colonies were formed after seeding the same number of peripheral blood nucleated cells from the age-matched, non-irradiated, healthy mice.

### Nicaraven significantly reduced DNA damage of cells in the bone marrow

**Figure 4:**
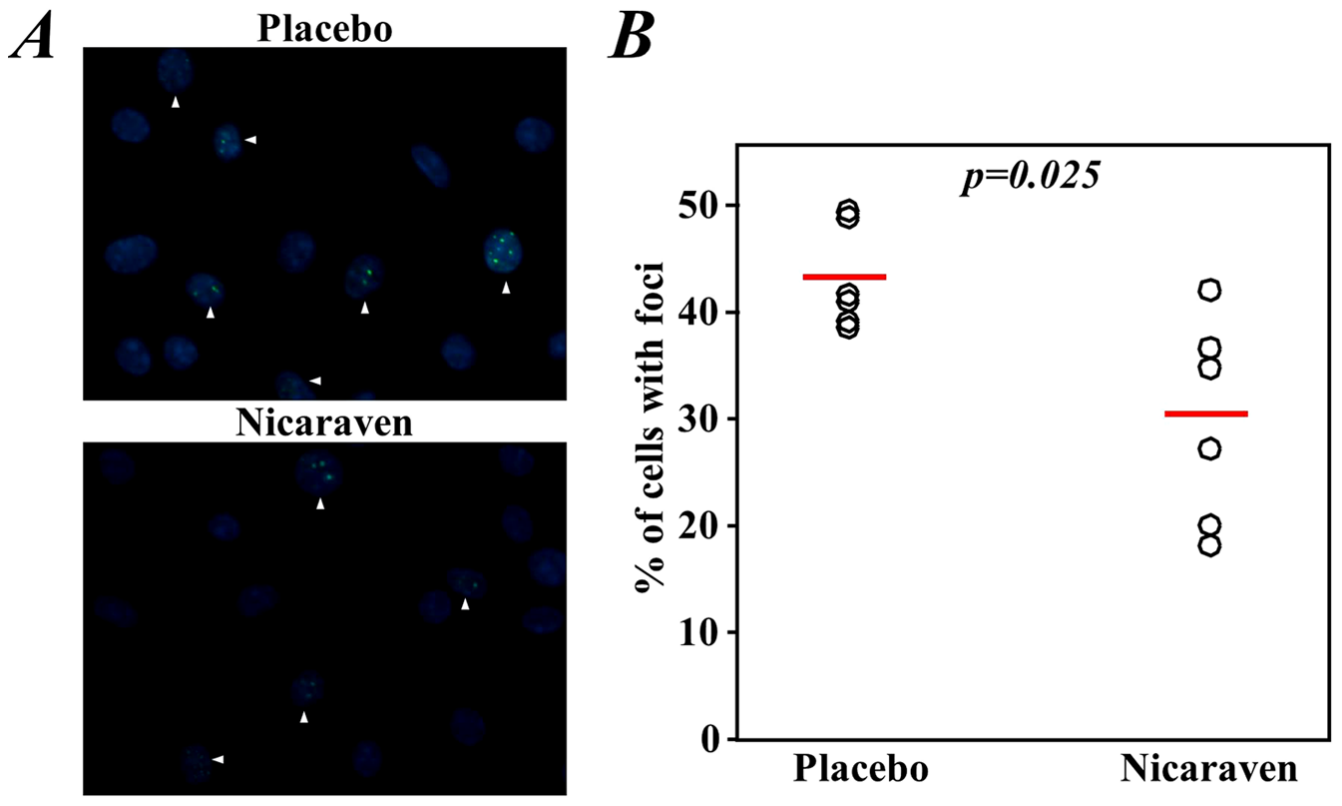
DNA damage in bone marrow cells. Bone marrow cells from mice were seeded in 4-well culture slides and cultured for 7 days. The DNA damage in the cells was estimated by immunostaining with an anti-53BP1 antibody. **A**) Representative images show the formation of 53BP1 foci within the nuclei of some cells (arrowheads). **B**) Quantitative analysis shows that the percentages of cells with 53BP1 foci were significantly lower in the Nicaraven group than the Placebo group. The open circles represent data from each mouse and the red lines indicate median values of each group.

We evaluated the DNA damage in the bone marrow cells based on counting the formation of 53BP1 foci in the nuclei by immunostaining analysis. A quantitative analysis showed that the percentages of cells with 53BP1 foci in the nuclei were significantly lower in the bone marrow cells collected from mice given nicaraven than those that received a placebo (*p = 0.025*, ***Fig. 4***). However, the percentages of cells with 53BP1 foci in both the nicaraven- and placebo-treated mice were much higher than that of the non-irradiated healthy mice (12.5±5.1%).

### Nicaraven did not significantly decrease the levels of intracellular and mitochondrial ROS

**Figure 5:**
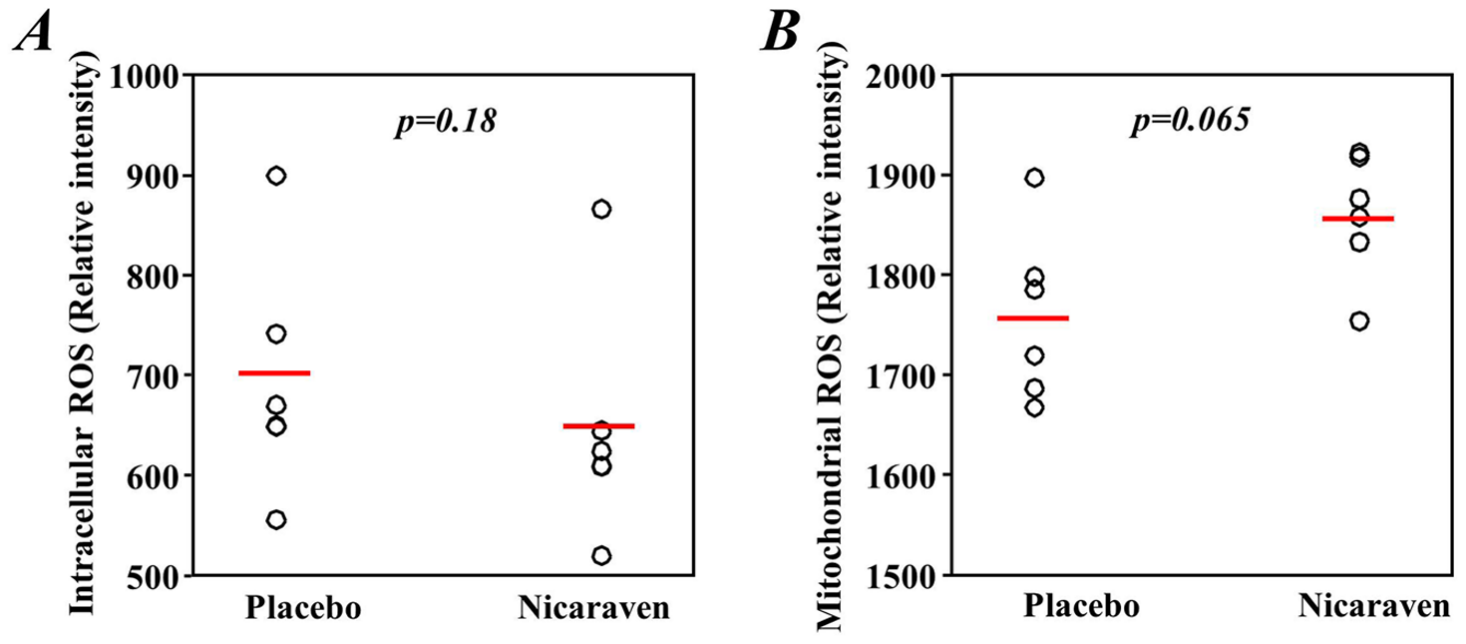
Intracellular and mitochondrial ROS in bone marrow cells. Mice were exposed daily to 1 Gy γ-rays for 5 days in succession, and either nicaraven or a placebo was given after each exposure. The cells were collected 2 days after the last radiation treatment and then loaded with 10 µM CM-H_2_DCFDA or 5 µM MitoSOX Red at 37°C for 30 minutes. The intracellular ROS (**A**) and mitochondrial ROS (**B**) were detected as the mean fluorescence intensity in all cells by flow cytometry. The open circles represent data from each mouse and the red lines indicate median values of each group.

As nicaraven is well recognized as a hydroxyl radical-specific scavenger, we measured the levels of intracellular and mitochondrial ROS in the bone marrow cells. The intracellular and mitochondrial ROS levels in the bone marrow cells obtained 2 days after the last radiation or drug treatment were unexpectedly similar between the nicaraven- and placebo-treated mice (***Fig. 5***).

### Nicaraven significantly decreased the urinary 8-OHdG

**Figure 6:**
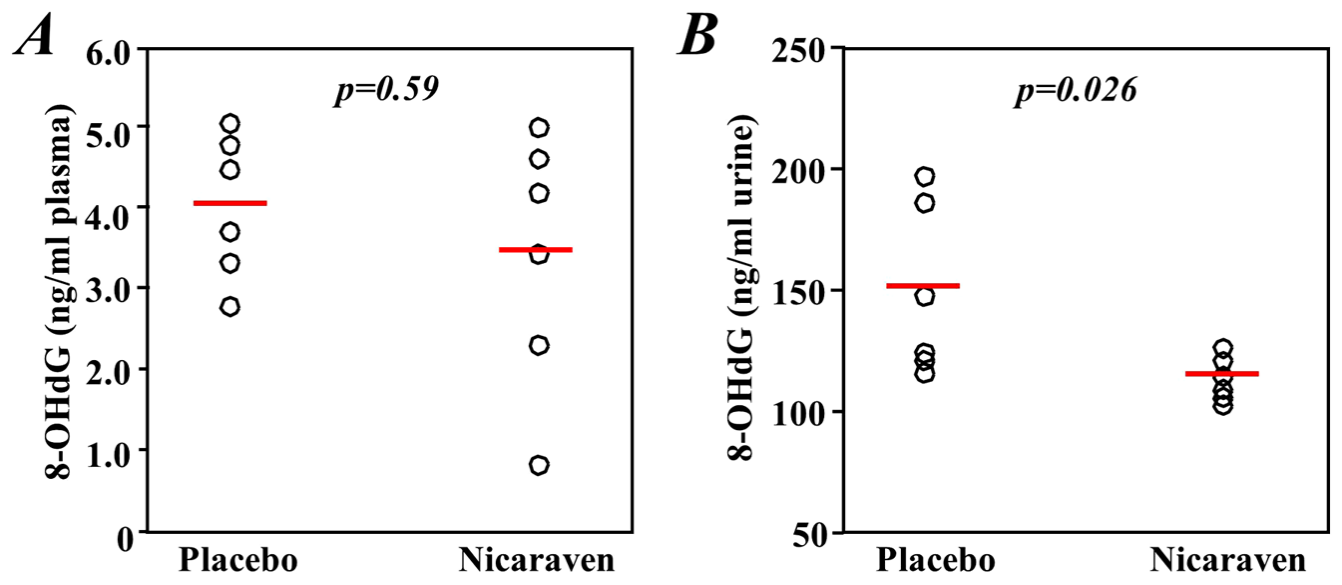
The levels of 8-OHdG in the plasma and urine. The plasma 8-OHdG levels were very low and did not significantly differ between the two groups (**A**), but the levels of urinary 8-OHdG were significantly lower in the mice that received nicaraven than in those that received a placebo (**B**). The open circles represent the mean of data from a mouse with duplicate assay. The red lines indicate median values of each group.

Low plasma levels of 8-OHdG were measured in both the nicaraven- and placebo-treated mice. Consequently, we found no significant difference between the two groups (p = 0.59, ***Fig. 6A***). In contrast, the urinary levels of 8-OHdG were significantly lower in the Nicaraven group than in the Placebo group 2 days after the last radiation and treatments (p = 0.026, ***Fig. 6B***). The 8-OHdG in plasma and urine of the non-irradiated healthy mice is not detectable.

### Nicaraven significantly decreased the levels of inflammatory cytokines in the plasma

**Figure 7:**
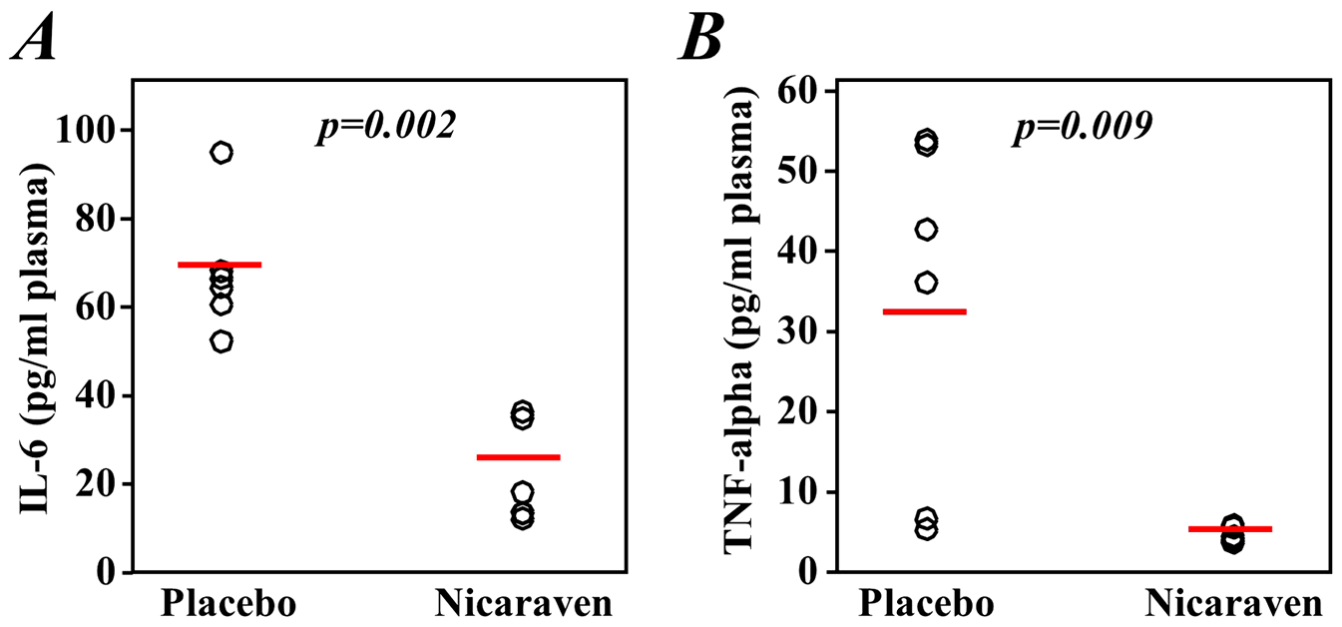
Inflammatory cytokines in the plasma. The plasma was collected 2 days after the last radiation treatment, and the levels of the inflammatory cytokines IL-6 (**A**) and TNF-α (**B**) in the plasma were measured by ELISA. The open circles represent the mean of data from a mouse with duplicate assay. The red lines indicate median values of each group.

As a robust release of various inflammatory cytokines is considered to indirectly contribute to radiation injury, we further investigated whether nicaraven can protect against radiation damage through the inhibition of the inflammatory responses after radiation exposure. We found that the levels of both IL-6 and TNF-α in the plasma were significantly decreased in mice that were given nicaraven compared with those that received a placebo (p = 0.002 and p = 0.009, ***Fig. 7***). Both IL-6 and TNF-α is not detectable in the plasma of the non-irradiated healthy mice by using the ELISA kit.

## Discussion

In addition to the direct induction of DNA double-strand breaks [1], ionizing radiation can trigger the releases of ROS and the production of a multitude of inflammatory cytokines, which indirectly contribute to the consequent damage to cells and tissues [2]–[10]. Therefore, the scavenging of ROS and the suppression of the inflammatory response are thought to be potential pharmacological interventions for mitigating radiation-induced injury. It has been demonstrated that the intake of anti-oxidants can attenuate radiation-induced injury [11]–[14], and amifostine, a ROS-scavenging drug, has been approved by the FDA as a cytoprotective adjuvant for cancer patients receiving radiotherapies [15]. However, the development of new protective and therapeutic drugs for ionizing radiation has been particularly emphasized in Japan since the Fukushima nuclear power plant accident took place.

Nicaraven, a chemically synthesized compound produced by Chugai Pharmaceutical Co Ltd., was originally recognized as a powerful radical scavenger that effectively protects various tissues or organs against injury, particularly ischemia-reperfusion injury in the brain [17]–[25]. Considering the well-recognized anti-oxidative properties and likely anti-inflammatory activities of nicaraven, we examined the protective effects of nicaraven on radiation injury. We found that post-radiation administration of nicaraven effectively improved the radiation-induced decreases in the number and colony-forming capacity of hematopoietic stem/progenitor cells, indicating that post-radiation administration of nicaraven may be beneficial for protecting against radiation-induced injury, even after high-dose exposures. The number and colony-forming capacity of hematopoietic stem/progenitor cells in mice that received nicaraven remained at relatively low levels (less than half of the levels in healthy, non-irradiated mice) 2 days after the last radiation treatment, indicating that the post-administration of nicaraven significantly mitigated, but did not completely prevent, radiation-induced injury after whole-body exposure to 1 Gy γ-rays daily for successive 5 days.

Regarding the defense mechanisms by which nicaraven protects against radiation-induced injury, we have found that nicaraven significantly decreased the urinary levels of 8-OHdG, indicating the effective mitigation of oxidative damage by the compound after radiation. The reason that no statistically significant difference was shown in the plasma concentrations of 8-OHdG between the placebo- and nicaraven-treated groups may be due to the relatively low levels of 8-OHdG obtained in the former group. However, the levels of ROS in the bone marrow cells did not differ between the mice that received either nicaraven or a placebo 2 days after the last radiation or drug treatment. This observation may be explained in terms of the following hypotheses. First, it is possible that the time point of measurement in this study was too late to precisely detect the difference in ROS levels between the two experimental groups. Otherwise, because the abrupt release of ROS was known to be brought about by various stimulations, the measured ROS levels in the isolated bone marrow cells in this study would not represent their real ROS levels within the bodies of mice because a robust release of ROS in the bone marrow cells might be induced by artificial factors during the measuring processes, including the mechanical stresses of cell collection and isolation, the changes of temperature (37°C in the body *vs.* 4°C in a test tube sometimes), and the different oxygen tensions (1∼3% O_2_ in bone marrow *vs.* 20% O_2_ in a test tube). Therefore, it remains unknown at the present time whether nicaraven decreases ROS in cells in the *in vivo* microenvironment, especially at an earlier phase after nicaraven administration, and answering this question requires further investigation. A recent study reported that increased ROS levels are not necessarily correlated with functional impairment of hematopoietic stem cells [33]. Taken together, the decreased levels of urinary 8-OHdG by nicaraven strongly suggest that the protective effects of nicaraven on radiation-induced injury may be related at least partly to its anti-oxidative properties.

Increasing evidence has shown that the release of a multitude of cytokines in response to radiation exposure can contribute to the damage to the cells/tissues [6]–[10]. As nicaraven has been found to reduce the infiltration of neutrophils into injured tissues [24], we wondered whether nicaraven could mitigate radiation-induced injury through the suppression of the inflammatory response. By measuring the levels of inflammatory cytokines, we found that the administration of nicaraven significantly decreased the levels of two major inflammatory cytokines, IL-6 and TNF-α, in the plasma of mice exposed to radiation. This observation suggests that nicaraven protects against radiation injury by inhibiting the release of inflammatory cytokines. However, further experiments would be needed to confirm the direct anti-inflammatory activity of nicaraven because the attenuation of radiation-induced injury through the scavenging of oxygen radicals by nicaraven may also be attributable to the decreased levels of inflammatory cytokines.

Of particular interest is the finding that the urine volume in the mice that received nicaraven was found to be more than double that of the mice that received placebo (data not shown). As nicaraven has previously been found to protect against ischemic injury of the kidney [22], we wondered whether any radiation-induced histological abnormalities in the kidney were mitigated by treatment with nicaraven. A simple hematoxylin and eosin staining did not demonstrate any obvious abnormalities within the kidney in mice that received either nicaraven or placebo (data not shown) 2 days after a total γ-ray exposure of 5 Gy (1 Gy/day for 5 days in succession). Although electron microscopy and immunohistological analyses may reveal certain pathological changes in the kidney, our speculation at the present time is that the higher urine volume obtained in the nicaraven-treated mice is related to the improvement of hypoperfusion of blood flow in the kidney [22], [34].

This study has several limitations. First, we only measured the percentages of c-kit- and CD34-positive cells in freshly-collected cells and counted the total formed number of all colonies after cell culture. It is still unclear whether the protective effect of nicaraven will be differed among different types of stem/progenitor cells, especially to identify the protective effect on the rare population of long-term hematopoietic stem cells (Lin−/Sca1+/c-Kit+, LSK cells). Second, although the protective effects of nicaraven against radiation-induced injury likely associated with the anti-oxidative and anti-inflammatory activities, further study will be needed to understand the relevant molecular mechanism by an *in vitro* approach. Otherwise, it is also very important to compare the protective effect against radiation injury between nicaraven and amifostine, a cytoprotective drug clinically used for radiotherapies [15].In summary, we have clearly demonstrated that the administration of nicaraven significantly increased the number, improved the colony-forming capacity, and diminished the DNA damage in hematopoietic stem/progenitor cells in mice after a total γ-ray exposure of 5 Gy. Furthermore, the mechanisms on the protective effects of nicaraven against radiation-induced injury are likely to be related at least partly to the anti-oxidative and anti-inflammatory properties of the compound. Many antioxidants have been shown to effectively attenuate radiation-induced injury [11]–[14]. Although we have not yet compared the protective potency of nicaraven on radiation injury with those of other antioxidants and drugs, nicaraven may be a potentially powerful new protective treatment for radiation-induced injury. As phase III studies of nicaraven for the treatment of acute cerebrovascular diseases were fully completed with an excellent safety profile [18], these clinical findings, as well as the experimental data shown in the present study, deserve further clinical investigation to ascertain the potential benefits of nicaraven for the treatment of radiation injury.

## References

[1] Huang L, Snyder A-R, Morgan W-F (2003) Radiation-induced genomic instability and its implications for radiation carcinogenesis. Oncogene 22: 5848–5854. 12947391 10.1038/sj.onc.1206697

[2] Robbins ME, Zhao W (2004) Chronic oxidative stress and radiation-induced late normal tissue injury: a review. Int J Radiat Biol 80: 251–259. 15204702 10.1080/09553000410001692726

[3] Ridley AJ, Whiteside JR, McMillan TJ, Allinson SL (2009) Cellular and sub-cellular responses to UVA in relation to carcinogenesis. Int J Radiat Biol 85: 177–195. 19296341 10.1080/09553000902740150

[4] Zhao W, Robbins ME (2009) Inflammation and chronic oxidative stress in radiation-induced late normal tissue injury: therapeutic implications. Curr Med Chem 16: 130–143. 19149566 10.2174/092986709787002790

[5] Wang Y, Liu L, Pazhanisamy SK, Li H, Meng A, et al (2010) Total body irradiation causes residual bone marrow injury by induction of persistent oxidative stress in murine hematopoietic stem cells. Free Radic Biol Med 48: 348–356. 19925862 10.1016/j.freeradbiomed.2009.11.005 PMC2818724

[6] Gallet P, Phulpin B, Merlin JL, Leroux A, Bravetti P, et al (2011) Long-term alterations of cytokines and growth factors expression in irradiated tissues and relation with histological severity scoring. PLoS One 6: e29399. 22216271 10.1371/journal.pone.0029399 PMC3245280

[7] Zhang M, Yin L, Zhang K, Sun W, Yang S, et al (2012) Response patterns of cytokines/chemokines in two murine strains after irradiation. Cytokine 58: 169–177. 22277799 10.1016/j.cyto.2011.12.023

[8] Cachaço AS, Carvalho T, Santos AC, Igreja C, Fragoso R, et al (2010) TNF-alpha regulates the effects of irradiation in the mouse bone marrow microenvironment. PLoS One 5: e8980. 20126546 10.1371/journal.pone.0008980 PMC2813873

[9] Rastogi S, Coates PJ, Lorimore SA, Wright EG (2012) Bystander-type effects mediated by long-lived inflammatory signaling in irradiated bone marrow. Radiat Res 177: 244–250. 22149991 10.1667/rr2805.1

[10] Ossetrova NI, Blakely WF (2009) Multiple blood-proteins approach for early-response exposure assessment using an in vivo murine radiation model. Int J Radia Biol 85: 837–850. 19863200

[11] Li H, Wang Y, Pazhanisamy SK, Shao L, Batinic-Haberle I, et al (2011) Mn(III) meso-tetrakis-(N-ethylpyridinium-2-yl) porphyrin mitigates total body irradiation-induced long-term bone marrow suppression. Free Radic Biol Med 51: 30–37. 21565268 10.1016/j.freeradbiomed.2011.04.016 PMC3390209

[12] Bairati I, Meyer F, Gélinas M, Fortin A, Nabid A, et al (2005) Randomized trial of antioxidant vitamins to prevent acute adverse effects of radiation therapy in head and neck cancer patients. J Clin Oncol 23: 5805–5813. 16027437 10.1200/JCO.2005.05.514

[13] Rhodes LE, Shahbakhti H, Azurdia RM, Moison RM, Steenwinkel MJ, et al (2003) Effect of eicosapentaenoic acid, an omega-3 polyunsaturated fatty acid, on UVR-related cancer risk in humans. An assessment of early genotoxic markers. Carcinogenesis 24: 919–925. 12771037 10.1093/carcin/bgg038

[14] Delanian S, Porcher R, Balla-Mekias S, Lefaix JL (2003) Randomized, placebo-controlled trial of combined pentoxifylline and tocopherol for regression of superficial radiation-induced fibrosis. J Clin Oncol 21: 2545–250. 12829674 10.1200/JCO.2003.06.064

[15] Bourhis J, Blanchard P, Maillard E, Brizel DM, Movsas B, et al (2011) Effect of amifostine on survival among patients treated with radiotherapy: a meta-analysis of individual patient data. J Clin Oncol 29: 2590–2597. 21576630 10.1200/JCO.2010.33.1454

[16] Akimoto T (2000) Quantitative analysis of the kinetic constant of the reaction of N, N′-propylenedinicotinamide with the hydroxyl radical using dimethyl sulfoxide and deduction of its structure in chloroform. Chem Pharm Bull (Tokyo) 48: 467–476. 10783063 10.1248/cpb.48.467

[17] Asano T, Johshita H, Koide T, Takakura K (1984) Amelioration of ischaemic cerebral oedema by a free radical scavenger, AVS: 1,2-bis(nicotinamido)-propane. An experimental study using a regional ischaemia model in cats. Neurol Res 6: 163–168. 6152308 10.1080/01616412.1984.11739683

[18] Asano T, Takakura K, Sano K, Kikuchi H, Nagai H, et al (1996) Effects of a hydroxyl radical scavenger on delayed ischemic neurological deficits following aneurysmal subarachnoid hemorrhage: results of a multicenter, placebo-controlled double-blind trial. J Neurosurg 84: 792–803. 8622153 10.3171/jns.1996.84.5.0792

[19] Imperatore C, Germano A, d'Avella D, Tomasello F, Costa G (2000) Effects of the radical scavenger AVS on behavioral and BBB changes after experimental subarachnoid hemorrhage. Life Sci 66: 779–790. 10698353 10.1016/s0024-3205(99)00651-7

[20] Asano T, Sasaki T, Koide T, Takakura K, Sano K (1984) Experimental evaluation of the beneficial effect of an antioxidant on cerebral vasospasm. Neurol Res 6: 49–53. 6147779 10.1080/01616412.1984.11739663

[21] Yokota R, Fukai M, Shimamura T, Suzuki T, Watanabe Y, et al (2000) A novel hydroxyl radical scavenger, nicaraven, protects the liver from warm ischemia and reperfusion injury. Surgery 127: 661–669. 10840362 10.1067/msy.2000.105864

[22] Masaki Y, Kumano K, Endo T, Iwamura M, Koshiba K, et al (1998) Protective effect of nicaraven against prolonged cold kidney preservation and reperfusion injury. Transplant Proc 30: 3758–3760. 9838646 10.1016/s0041-1345(98)01223-8

[23] Alam MS, Ku K, Yamauchi M, Hashimoto M, Nosaka S, et al (1998) Protective effects of nicaraven, a new hydroxyl radical scavenger, on the endothelial dysfunction after exposure of pig coronary artery to hydroxyl radicals. Mol Cell Biochem 178: 237–243. 9546605 10.1023/a:1006855917392

[24] Zingarelli B, Scott GS, Hake P, Salzman AL, Szabo C (2000) Effects of nicaraven on nitric oxide-related pathways and in shock and inflammation. Shock 13: 126–134. 10670842 10.1097/00024382-200013020-00006

[25] Masana Y, Yoshimine T, Fujita T, Maruno M, Kumura E, et al (1995) Reaction of microglial cells and macrophages after cortical incision in rats: effect of a synthesized free radical scavenger, (+/−)-N,N′-propylenedinicotinamide (AVS). Neurosci Res 23: 217–221. 8532218 10.1016/0168-0102(95)00936-n

[26] Mori Y, Takashima H, Seo H, Ohkawa M, Yamamoto G, et al (1993) Experimental studies on nicaraven as a radioprotector: survival ratio of mice, spleen colony formation, blood picture and lipid peroxidation. Okayama Ishi 105: 673–680.

[27] Mori Y, Takashima H, Seo H, Yamamoto G, Liu J, et al (1993) Experimental studies on nicaraven as radioprotector–free radical scavenging effect and the inhibition of the cellular injury. Nihon Igaku Hoshasen Gakkai Zasshi 53: 704–712. 8337113

[28] Watanabe M, Akiyama N, Sekine H, Mori M, Manome Y (2006) Inhibition of poly (ADP-ribose) polymerase as a protective effect of nicaraven in ionizing radiation- and ara-C-induced cell death. Anticancer Res 26: 3421–3427. 17094462

[29] Yoshida T, Goto S, Kawakatsu M, Urata Y, Li TS (2012) Mitochondrial dysfunction, a probable cause of persistent oxidative stress after exposure to ionizing radiation. Free Radic Res 46: 147–153. 22126415 10.3109/10715762.2011.645207

[30] Li TS, Ikeda S, Kubo M, Ohshima M, Kurazumi H, et al (2011) Diabetic impairment of c-kit^+^ bone marrow stem cells involves the disorders of inflammatory factors, cell adhesion and extracellular matrix molecules. PLoS One 6: e25543. 21984919 10.1371/journal.pone.0025543 PMC3184966

[31] Li TS, Marbán E (2010) Physiological levels of reactive oxygen species are required to maintain genomic stability in stem cells. Stem Cells 28: 1178–1185. 20506176 10.1002/stem.438 PMC3408073

[32] Takemoto Y, Li TS, Kubo M, Ohshima M, Ueda K, et al (2011) Operative injury accelerates tumor growth by inducing mobilization and recruitment of bone marrow-derived stem cells. Surgery 149: 792–800. 21507448 10.1016/j.surg.2011.02.005

[33] Merchant AA, Singh A, Matsui W, Biswal S (2011) The redox-sensitive transcription factor Nrf2 regulates murine hematopoietic stem cell survival independently of ROS levels. Blood 118: 6572–6579. 22039262 10.1182/blood-2011-05-355362 PMC3242719

[34] Yamamoto S, Teng W, Nishizawa S, Kakiuchi T, Tsukada H (2000) Improvement in cerebral blood flow and metabolism following subarachnoid hemorrhage in response to prophylactic administration of the hydroxyl radical scavenger, AVS, (+/−)-N,N′-propylenedinicotinamide: a positron emission tomography study in rats. J Neurosurg 92: 1009–1015. 10839263 10.3171/jns.2000.92.6.1009

